# Professional training and research: which resources?

**DOI:** 10.1186/1824-7288-41-S2-A41

**Published:** 2015-09-30

**Authors:** Lucia Guidarelli

**Affiliations:** 1 Ministry of Health, Rome, Italy

## 

The National Plan of Prevention (PNP 2014-2018) [1], which is the key document for the Italian Public Health, underlines the need to realize for all people an empowerment level able to increase or maintain the health control. At international level, the Ljubljana Charter [2] contains the sanitarian systems commitment to rearrange, in the aim to improve continually the quality, with reference to, in particular, the rate cost /efficacy of the system. The manual for medical practitioners and pediatricians (Ministry of Heath, 2010) [3] acknowledges the seven fields of skills for operators, as defined by the Canadian Patient Safety Institute, the CPSI.

Continuous training for health professionals started as a national program in Italy in 2002; some years later (2007) administrative control had been transferred from the Ministry of Health to the National Agency for Health Services, Agenas. At distance training and online updating are now becoming very common, close to traditional kinds of events for training, always in respect of the Evidence Based Medicine (EBM).

We can identify, in the continuous sanitarian training system, different roles and various kinds of providers. Relevant organisations include the National Committee for Training, in the Ministry of Health, the Warranty Committee, and the National Observatory about Quality of Training.

Regarding research in the field of health, we can consider it as the architrave of the National Health Service; the Report on Health Status in Italy 2012-13 [4] defines some essential aspects of it, such as the improvement of criteria of selection of the projects, to simplify bureaucratic procedures, evaluation and the rapid spread of the results.

The National Committee for Sanitarian Research defines the program and initiatives, monitors and evaluates the results. Research institutes involved in Italy must accept different challenges, according with the Singapore Declaration [5], which defined some criteria about loyalty and professional honesty.

The European Union invested 80M Euro in research with Horizon 2020, in the aim of destroying barriers and realising, all over the world, a common environment concerning knowledge, research and innovation.

A recent piece of research related to the food habits in the age of complementary feeding is Nutrintake 6/36 [6], the first Italian study which confirms some excesses and deficiencies in the diet. It suggests the opportunity of the definition of specific nutritional guidelines.
